# Bacteriophage vB_SepP_134 and Endolysin LysSte_134_1 as Potential Staphylococcus-Biofilm-Removing Biological Agents

**DOI:** 10.3390/v16030385

**Published:** 2024-02-29

**Authors:** Natalia N. Golosova, Andrey L. Matveev, Nina V. Tikunova, Yana A. Khlusevich, Yulia N. Kozlova, Vera V. Morozova, Igor V. Babkin, Tatiana A. Ushakova, Elena V. Zhirakovskaya, Elizaveta A. Panina, Elena I. Ryabchikova, Artem Y. Tikunov

**Affiliations:** Institute of Chemical Biology and Fundamental Medicine, Siberian Branch of Russian Academy of Sciences, 630090 Novosibirsk, Russia; n.golosova@g.nsu.ru (N.N.G.); tikunova@niboch.nsc.ru (N.V.T.); khlusevichjana@mail.ru (Y.A.K.); ulona79@mail.ru (Y.N.K.); morozova@niboch.nsc.ru (V.V.M.); i_babkin@mail.ru (I.V.B.); ushakova@niboch.nsc.ru (T.A.U.); e.panina@g.nsu.ru (E.A.P.); lenryab@niboch.nsc.ru (E.I.R.)

**Keywords:** bacteriophage, endolysin, *Staphylococcus epidermidis*, *Staphylococcus aureus*, antimicrobial agent, biofilm

## Abstract

Bacteria of the genus *Staphylococcus* are significant challenge for medicine, as many species are resistant to multiple antibiotics and some are even to all of the antibiotics we use. One of the approaches to developing new therapeutics to treat staphylococcal infections is the use of bacteriophages specific to these bacteria or the lytic enzymes of such bacteriophages, which are capable of hydrolyzing the cell walls of these bacteria. In this study, a new bacteriophage vB_SepP_134 (St 134) specific to *Staphylococcus epidermidis* was described. This podophage, with a genome of 18,275 bp, belongs to the *Andhravirus* genus. St 134 was able to infect various strains of 12 of the 21 tested coagulase-negative *Staphylococcus* species and one clinical strain from the *Staphylococcus aureus* complex. The genes encoding endolysin (LysSte134_1) and tail tip lysin (LysSte134_2) were identified in the St 134 genome. Both enzymes were cloned and produced in *Escherichia coli* cells. The endolysin LysSte134_1 demonstrated catalytic activity against peptidoglycans isolated from *S. aureus, S. epidermidis*, *Staphylococcus haemolyticus*, and *Staphylococcus warneri*. LysSte134_1 was active against *S. aureus* and *S. epidermidis* planktonic cells and destroyed the biofilms formed by clinical strains of *S. aureus* and *S. epidermidis*.

## 1. Introduction

In recent decades, the treatment of patients with bacterial infections has often been complicated by the multiple drug resistance (MDR) of their causative agents. Among these causative agents, the World Health Organization (WHO) has identified 12 pathogens that pose a global threat to human health [1]. This list includes ESKAPE pathogens and identifies three categories of pathogens depending on their danger and the urgency of developing new antibacterial drugs. *Staphylococcus aureus* strains, which are resistant to methicillin and vancomycin (MRSA and VRSA), were assigned to the high-priority group [2]. Given the increasing incidence of MDR among nosocomial strains of *Staphylococcus epidermidis* [3] and their ability to form biofilms [4,5,6], an assessment of the antibacterial activity of new drugs against both *S. aureus* and *S. epidermidis,* and other coagulase-negative staphylococci is required.

Among the promising therapeutic agents that may become an alternative or addition to antibiotics are bacteriophages. These viruses can specifically infect a bacterial pathogen and eliminate it from an organism. In most cases, phages are safe for the patient’s microbiota and, after the elimination of the target pathogen, are excreted naturally without disrupting the functioning of the excretory and immune systems [7,8,9]. Phages can be used to treat infants, the elderly, and even cancer patients [10,11,12]. Despite many advantages, the use of bacteriophages has a number of limitations [13,14], which determine certain requirements for the bacteriophages used. Thus, only phages with a proven inability to adopt a lysogenic lifestyle and with the absence of undesirable genes in their genome can be used in phage therapy. In addition, the phages used should have high lytic activity and at least two phages that are active against the pathogen should be used to avoid the development of phage resistance [11,15]; another approach is to replace therapeutic phages during treatment if resistance occurs [16]. Moreover, the typical high specificity of phages requires the selection of personalized phages in each case; otherwise, polyvalent phage cocktails are used. However, re-administration of the same polyvalent cocktail to the same patient to treat another infection increases its effect on the immune system [17], and the efficacy of the cocktail can be reduced [18,19]. So, strictly lytic phages with a broad host range and a thoroughly annotated genome without toxin genes are preferred for phage therapy [20].

Phage enzymes capable of destroying bacterial cell walls have advantages over bacteriophages [21,22,23]. These lytic enzymes have a wider host spectrum than their parental phages and bacteria do not acquire resistance to them; in addition, these lytic proteins demonstrate clear bioavailability and pharmacokinetics [24]. Importantly, standardized biotechnological methods can be used for their production. The WHO has recently recognized phage lytic enzymes as promising therapeutics [25].

There are two large groups of phage lytic enzymes: structural lysins, also known as tail-associated murein lytic enzymes (TAME) or tail-associated lysins, which ensure the penetration of phage DNA into the cell after phage adsorption at the beginning of the phage infection cycle; and endolysins, which are produced inside infected cells generating the release of new phage particles at the end of the infection cycle [26,27]. Notably, endolysins can hydrolyze the peptidoglycans of the cell wall from outside. This can be used, in particular, against Gram-positive pathogens and the biofilms formed by them. The endolysins of phages specific to Gram-positive bacteria usually have one or two enzymatically active domains (EAD) and a cell-binding domain (CBD). Their CBD can be C-terminal or it can be located between two EADs. The potential efficacy of the endolysins of *Staphylococcus* phages has been demonstrated [24,28,29,30,31,32].

In this study, the *S. epidermidis* phage vB_SepP_134 (St 134) was isolated and characterized. This podophage has a genome of 18,275 bp and was able to infect more than ten coagulase-negative *Staphylococcus* species and one strain from the *Staphylococcus aureus* complex. Two genes encoding different endolysins were found in the St 134 genome and one endolysin was able to destroy peptidoglycans from the cell walls of *S. aureus* and *S. epidermidis,* as well as their biofilms.

## 2. Materials and Methods

### 2.1. Bacterial Strains and Growth Conditions 

A total of 457 *Staphylococcus* strains (24 species) from the Collection of Extremophile Microorganisms and Type Cultures (CEMTC) of ICBFM SB RAS were used to test the host range of St 134. These strains have been collected since 2009 from samples obtained from hospitalized patients and outpatients (from different hospitals and hospital departments). Among the strains used, none were derived from outbreaks of staphylococcal infection. Each isolate was analyzed using microscopy. *Staphylococcus* species were determined by sequencing their 16S rRNA gene fragment (~1400 bp); their antibiotic resistance had been previously tested using the disk-diffusion method (Oxoid™, Thermo Fisher Scientific, Waltham, MA, USA) in accordance with the EUCAST recommendations [3]. A basic description of the *Staphylococcus* strains which were sensitive to St 134 is provided in Table S1. In addition, *S. aureus* CEMTC 675, *S. aureus* CEMTC 1685, *S. epidermidis* CEMTC 2058, and several other coagulase-negative strains, were used to investigate the antibacterial activity of the recombinant phage endolysin and tail tip lysin. All bacterial strains were cultivated in Luria Bertani (LB) media/agar (1.5% *w*/*v*) (10 g/L peptone, 5 g/L yeast extract, 10 g/L NaCl, pH: 7.0, Becton Dickinson, Franklin Lakes, NJ, USA). The planktonic bacterial cells were grown in an orbital shaker incubator at 150 rpm and 37 °C (Infors HT, Bottmingen, Switzerland). 

### 2.2. Phage St 134 Isolation and Propagation

Phage St 134 was found in a smear taken from the armpit of an outpatient with psoriasis. To isolate the phage, 1 mL of sterile phosphate-buffered saline (PBS), pH 7.5, was added to a swab and the obtained suspension was sterilized through a 0.22 μm filter (Wuxi Nest Biotechnology, Wuxi, China). Drops of the filtrate were placed onto freshly prepared lawns of various bacterial cultures, from CEMTC, in top agar (Becton, Dickinson and Company Sparks Difco Laboratories, Franklin Lakes, NJ, USA). After overnight incubation at 37 °C, phage plaques were found on the Petri dish containing *S. epidermidis* CEMTC 2058, which was considered the host strain. This strain was isolated from a purulent wound several months before phage selection and kindly provided to us by our colleagues from the Novosibirsk Research Institute of Traumatology and Orthopedics. 

The obtained spot was cut from the agar, suspended in sterile PBS, and incubated with shaking overnight to extract phage particles from the agar. Then, serial ten-fold dilutions were dropped onto a fresh layer of *S. epidermidis* CEMTC 2058 to obtain single plaques. The procedure for single plaques was repeated four times. Both the samples that contained phage and the host strain were collected during a study that was approved by the Ethics Committee of the Novosibirsk Research Institute of Traumatology and Orthopedics (protocol 014/17-2 from 25 November 2011). Both patients whose samples were used to isolate the phage St 134 and its host strain provided informed consent.

To propagate the isolated phage St 134, exponentially growing in LB, *S. epidermidis* CEMTC 2058 was infected with St 134 and incubated with shaking at 37 °C until bacterial lysis occurred. The obtained bacterial lysate was clarified by centrifugation at 10,000× *g* for 30 min and phage particles were isolated using precipitation with polyethylene glycol (PEG) 6000 (AppliChem, Darmstadt, Germany), supplemented with NaCl, and subsequent centrifugation. Phage precipitate was dissolved in a sterile STM buffer (0.59 g of NaCl; 7.88 g of Tris-HCl, pH 7.5; and 2.38 g of MgCl_2_ per 1 L).

### 2.3. Electron Microscopy

Phage particles were visualized using transmission electron microscopy (TEM). A drop of purified phage St 134 (10^9^ pfu/mL) was adsorbed for 1 min on a copper grid covered with formvar film. Uranyl acetate was used as a contrast agent for the treatment of the grid covered with St 134 phage particles. TEM imaging was performed using a TEM JEM 1400 (JEOL, Tokyo, Japan). To obtain digital pictures a Veleta digital camera (Olympus SIS, Münster, Germany) was used. The average capsid size and tail length were calculated using four viral particles.

### 2.4. Biological Properties and the Host Range

To evaluate the biological properties of the St 134 phage, *S. epidermidis* CEMTC 2058 was used. All experiments were repeated twice, and each experiment had three replicates. Experiments on phage adsorption rate and burst size were carried out according to [33,34], with minor modifications [35]. To determine the adsorption time, an exponentially growing host cell culture (10^8^ CFU/mL) was infected with 10^5^ pfu/mL of St 134 (multiplicity of infection, MOI = 0.001). The infected culture was incubated with shaking at 37 °C and aliquots were taken every two minutes to determine the titer of free phages via immediate titration. For the one-step growth experiment, 10 mL of exponentially growing host cells were pelleted at 8000× *g* for 10 min and suspended in 0.5 mL of LB medium. Phage St 134 was added to the cells with a MOI of 0.001; the suspension was incubated for 5 min without shaking at 37 °C, and the cells were centrifuged. To discard non-adsorbed phage particles, the supernatant was carefully removed, and then the bacterial pellet was resuspended in 10 mL of LB medium. The infected culture was incubated with shaking at 37 °C; aliquots were taken and titrated immediately. The lytic activity of the St 134 phage was assessed as described previously [28], with modifications. Phage St 134 (10^7^ pfu/mL) was mixed with an *S. epidermidis* CEMTC 2058 culture that was in the exponential growth phase, with a MOI of 0.1, and incubated without shaking at 37 °C for 30 min to improve infection. Then, the infected culture was incubated with shaking and aliquots were taken every 60 min, diluted, seeded on the LB agar, and incubated overnight at 37 °C. The bacterial killing curve was evaluated based on the counted CFU. To determine the host range of the St 134 phage, a spot assay [35] was carried out using >450 strains belonging to 24 *Staphylococcus* species. When a spot was observed, serial ten-fold dilutions of the ST 134 suspension were dropped onto a fresh layer of the corresponding strain to obtain plaques.

### 2.5. Phage DNA Purification and Complete Genome Sequencing

Genome DNA was isolated from a St 134 stock (10^9^ PFU/mL) as described previously [36]. Phage particles were precipitated using a PEG/NaCl solution and suspended in a STM buffer (10 mM NaCl, 50 mM Tris-HCl, pH 8.0, 10 mM MgCl_2_). The obtained phage suspension was supplemented with 10–25 units of DNase I (Thermo Fisher Scientific, Waltham, MA, USA) in DNase I buffer (10 mM tris-HCl pH 7.5, 2.5 mM MgCl_2_, 0.1 mM CaCl_2_) and up to 10–20 µg/mL of RNase (Thermo Scientific), and incubated for 30 min at 37 °C. Then, SDS (final concentration of 0.5%); up to 20 mM of 0.5 M EDTA, pH 8.0; and Proteinase K (up to 100–200 µg/mL) were added, followed by incubation at 55 °C for 2–3 h or at 37 °C overnight. Phage DNA was purified using phenol chloroform extraction with subsequent ethanol precipitation. A paired-end library of phage St 134 DNA was prepared using the NEBNext Ultra II DNA Library Prep Kit from Illumina (New England Biolabs, Inc., Ipswich, MA, USA). Sequencing was carried out using the MiSeq Benchtop Sequencer and MiSeq Reagent Kit v.2 (2 × 250 base reads). The genome was assembled de novo by SPAdes v.3.15.4 and resulted in one genomic contig with an average coverage of 152. The St 134 phage genome was submitted to GenBank (http://www.ncbi.nlm.nih.gov, accessed on 15 May 2020) under the accession number KY471386.

### 2.6. Comparative Analysis of the Phage St 134 Genome and Proteome

A linearized St 134 genome sequence was imported into the online RAST server v. 2.0 [28] to identify the putative ORFs. The functions of the identified ORFs were predicted by BLASTP (NCBI, Bethesda, MD, USA), InterProScan, and HHpred algorithms. Predictions on the architecture of their domains and functional motives were made using CD-BLAST (NCBI, Bethesda, MD, USA). In addition, the results of the annotation were manually verified by checking all of the predicted proteins against the NCBI GenBank protein database (https://www.ncbi.nlm.nih.gov, accessed on 19 September 2022). 

To compare the St 134 proteome, ViPTree analysis with default parameters [37] was computed by tBLASTx based on genome-wide sequence similarities. The ViPTree version 3.7 web server was used (https://www.genome.jp/viptree, accessed on 3 August 2023). Phylogenetic analysis of St 134 proteins was performed using Maximum Likelihood trees. Protein sequences were extracted from the NCBI’s protein database using St 134 proteins as the query in a BLASTp search. Amino acid sequences were aligned using M-Coffee in the T-Coffee program [38]. Phylogenetic trees were constructed by the IQ-tree program v. 2.0.5 [39]; the best fit substitution model was applied according to ModelFinder [40]. Branch support was evaluated using 1000 ultrafast bootstrap replicates [41]. All trees were midpoint rooted and visualized in FigTree v1.4.1.

### 2.7. In Silico Identification and Structural Analysis of Putative Phage Lytic Enzymes

Lysins LysSte134_1 (GenBank: AQT25388) and LysSte134_2 (GenBank: AQT25384) were predicted using the Protein Database from NCBI (https://www.ncbi.nlm.nih.gov/protein, accessed on 15 January 2018) and the InterProScan software package (https://www.ebi.ac.uk/interpro, accessed on 15 January 2018). To calculate the solubility of LysSte134_1 and LysSte134_2, Protein-sol [42] (https://protein-sol.manchester.ac.uk, accessed on 15 October 2023) and SoluProt [43] (https://loschmidt.chemi.muni.cz/soluprot, accessed on 15 October 2023) were used. The assumed three-dimensional (3D) structures of the endolysin LysSte134_1 and tail tip lysin LysSte134_2 were predicted using AlphaFold2 [44] (https://colab.research.google.com/github/sokrypton/ColabFold/blob/main/AlphaFold2.ipynb, accessed on 21–24 April 2023). To visualize ribbon and surface representations of the studied proteins, the UCSF Chimera molecular visualizer, version 1.15 [45] was used.

### 2.8. Cloning, Expression, and Purification of Recombinant Lytic Enzymes

The genes encoding the LysSte134_1 and LysSte134_2 lytic enzymes were obtained by PCR using St 134 genomic DNA as a matrix and pairs of primers: endolysin_StE134_1U/endolysin_StE134_1L and endolysin_ste134_2U/endolysin_StE134_2L, respectively (Table 1). The expressing plasmid pQE-60 was cleaved using *Nco*I and *Bam*HI (Sibenzyme, Novosibirsk, Russia) and ligated with the obtained PCR fragments that were *Nco*I/*Bam*HI-digested. The obtained plasmids pQE-60/LysSte134_1 and pQE-60/LysSte134_2 were used to transform *Escherichia coli* XL1blue cells. Transformed cells were grown on LB agar with 50 μg/mL ampicillin at 37 °C overnight. To search for *E. coli* clones containing the corresponding expression plasmids, PCR was performed using the primers pQE60-SeqU and pQE60_SeqL (Table 1). Positive clones were sequenced by Sanger sequencing using a 3500 Genetic Analyzer (Thermo Fisher Scientific, Waltham, MA, USA).

To optimize the expression of recombinant enzymes, *E. coli* M15 cells were transformed with pQE-60/LysSte134_1 or pQE-60/LysSte134_2 plasmids. *E. coli* M15-pQE-60/LysSte134_1 or *E. coli* M15-pQE-60/LysSte134_2 cells were grown on LB agar with 50 μg/mL ampicillin at 37 °C overnight. To produce the recombinant proteins LysSte134_1 and LysSte134_2, fresh colonies of *E. coli* M15-pQE-60/LysSte134_1 or *E. coli* M15-pQE-60/LysSte134_2 were grown in LB medium with 50 μg/mL ampicillin until they reached OD_600_ 0.6–0.7. Then, 0.1 mM isopropyl-β-d-1-thiogalactopyranoside (IPTG) was added and cell cultures were grown overnight, with shaking at 150 rpm, at 12 °C, 20 °C, 30 °C, and 37 °C. Cells were centrifuged for 10 min at 6000× *g*, resuspended in 50 mM Tris-HCl pH 8.0, and lysed by sonication using an ultrasonic Sonopuls HD 2070 homogenizer (Bandelin, Mecklenburg, Vorpommern, Germany). The expression level of recombinant proteins and their localization were analyzed using polyacrylamide gel (PAGE) with 12.5% SDS. 

The recombinant LysSte134_1 protein was purified from the cytoplasm using Ni–NTA Sepharose chromatography (Qiagen, Venlo, Netherlands) according to the manufacturer’s instructions. LysSte134_1 was eluted with buffer A (50 mM NaH_2_PO_4_, 300 mM NaCl, 5 mM Tris-HCl, pH 8.0), containing an additional 100 mM of imidazole, and concentrated by an Amicon Ultra-4 filter (Millipore, Burlington, MA, USA) with a cutoff threshold of 10 kDa. The buffer was replaced with a storage buffer (S-buffer, 50 mM Tris-HCl pH 7.5, 300 mM NaCl) via dialysis. The concentration of purified recombinant endolysin was assessed using the Qubit protein assay kit (Thermo Fisher Scientific, Waltham, MA, USA) on a Qubit 4 fluorometer (Thermo Fisher Scientific, Waltham, MA, USA). The purity of the protein was quantified using a gel imager Gel Doc XR+ (Bio-Rad, Hercules, CA, USA) and software ImageLab 3.0 (Bio-Rad, Hercules CA, USA).

### 2.9. Zymography with Peptidoglycans from Staphylococcal Cells

Peptidoglycans from the cell walls of staphylococci were obtained in accordance with a previously described method [46]. A fresh overnight culture of *Staphylococcus* spp. cells diluted 500 times and cultured to OD600 = 1–1.5 in 1 L of LB medium at 37 °C, 150 rpm. The cells were centrifuged for 10 min at 6000× *g* or 8000× *g* (depending on the *Staphylococcus* species), resuspended in 10 mL of 4 M LiCl, boiled in a water bath for 20 min, and re-centrifuged. The precipitate was resuspended in mQ water, disrupted by ultrasonication (35% power) for 25 min using a Sonopuls HD 2070 homogenizer (Bandelin, Berlin, Germany), and centrifuged (12,000× *g* for 12 min). The obtained precipitate was resuspended in 12 mL of 4% SDS, boiled for 20 min, and pelleted by centrifugation (12 min at 12,000× *g*). The precipitate was resuspended in 12 mL of 1 M NaCl and pelleted by centrifugation (12 min, 12,000× *g*). This stage was repeated until the peptidoglycan precipitate became colorless. The resulting peptidoglycan precipitate was suspended in 1–2 mL of mQ water, re-centrifuged, dissolved in 2 mL of mQ water with 0.02% NaN3, and stored at a temperature of 4 °C. The concentration of peptidoglycan was determined using a SmartSpec Plus spectrophotometer (BioRad, Hercules, CA, USA). OD_540_ = 1.0 approximately corresponded to a concentration of peptidoglycan of 1 mg/mL. To assess their hydrolytic activity, recombinant enzymes were fractionated by electrophoresis using SDS-PAGE and purified peptidoglycans at a concentration of 0.1 mg/mL. After electrophoresis, the gel was gently washed with deionized water several times and transferred to a renaturing buffer containing 25 mM Tris–HCl, pH 7.2, and 1% Triton X-100. After incubation at 37 °C for 1 h, the gel was washed several times with deionized water and stained with 1% methylene blue solution. All zymographic experiments were repeated at least twice.

### 2.10. Antimicrobial Activity Assay

The ability of LysSte134_1 to destroy *Staphylococcus* cells was measured by counting the number of colony forming units (CFUs) after incubating them with the enzyme. Each *Staphylococcus* strain was cultured to its exponential phase (OD600 = 0.5) and centrifuged at 4000× *g* for five minutes. The harvested cells were washed and suspended in an R buffer (50 mM Tris-HCl; pH 8.0); the cell suspension was diluted to a titer of 10^7^ CFU/mL. Then, LysSte134_1 was diluted in R buffer or R buffer containing CaCl_2_, MgSO_4_, ZnCl_2_, or NiSO_4_ at concentrations of 1 μM or 5 μM. Cells without LysSte134_1 were used as a control. A total of 100 µL of cell suspensions were mixed with serial dilutions of LysSte134_1 in 96-well plates and incubated at 37 °C. Aliquots of these suspensions were plated on LB agar plates; colonies were counted the next day.

### 2.11. Biofilm Assay 

To obtained biofilms, staphylococcal cells (2 × 10^8^ CFU) were resuspended in 200 µL of sterile 0.9% NaCl, mixed with 10 mL of LB medium, and placed in a Petri dish with a pre-sterilized coverslip. The cells were incubated for 5 days at 37 °C. On the fifth day, their biofilm was evaluated using a Zeiss Axio Imager A2 microscope (Carl Zeiss, Oberkochen, Germany). The formed biofilms were treated with purified endolysin at a concentration of 25 µg/mL in S-buffer. S-buffer without endolysin was used as a negative control. Treated biofilms were incubated at 37 °C for 30 min and for 3 h after staining with 0.1% methyl violet. 

### 2.12. Statistical Analyses

Statistical analysis was carried out by a one-way analysis of variance (ANOVA) using Statistica 10 software (StatSoft Inc., Tulsa, OK, USA). The differences between the groups were considered significant at *p* < 0.05.

## 3. Results

### 3.1. St 134 Isolation, Plaques, and Phage Particles’ Morphology

Phage St 134 and its bacterial host strain were isolated from different patients. Phage St 134 was found in a skin wash sample that was obtained from an outpatient with psoriasis. The clinical strain *S. epidermidis* CEMTC 2058 that was used as the bacterial host had been previously isolated from a purulent wound and kindly provided by our colleagues from the Novosibirsk Research Institute of Traumatology and Orthopedics. Its taxonomy was confirmed by the sequencing of its 16S rRNA gene (GenBank ID OP393915). On a fresh lawn of the host strain, the phage St 134 formed cloudy plaques with a diameter of 0.8–1 mm.

**Figure 1:**
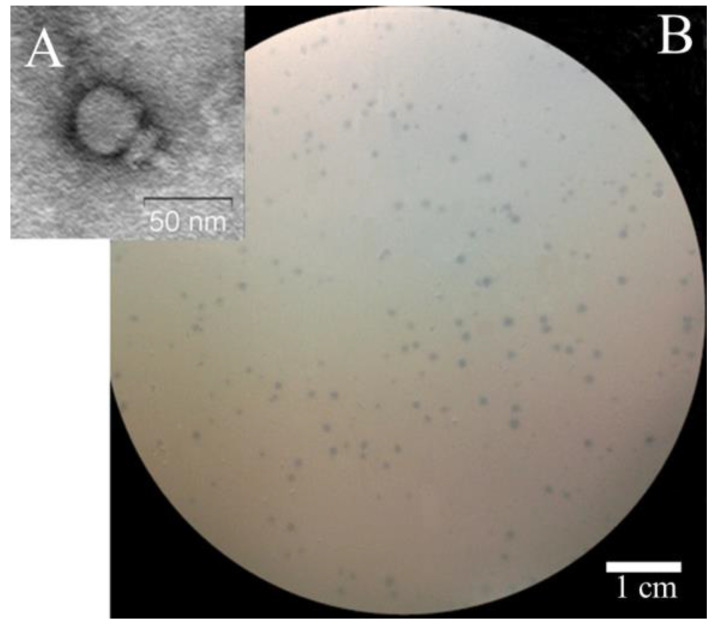
Electron micrograph of St 134 phage particle negatively stained with 1% uranyl acetate (**A**) and phage St 134 plaques on a lawn of *S. epidermidis* CEMTC 2058 (**B**).

Electron microscopy indicated that St 134 is a podophage with an icosahedral head (Ø 38.7 ± 0.7 nm) connected to a short (37.9 ± 1.6 nm) non-contractile tail (Figure 1 and Figure S1). 

### 3.2. St 134′s Biological Properties and Host Range

**Figure 2:**
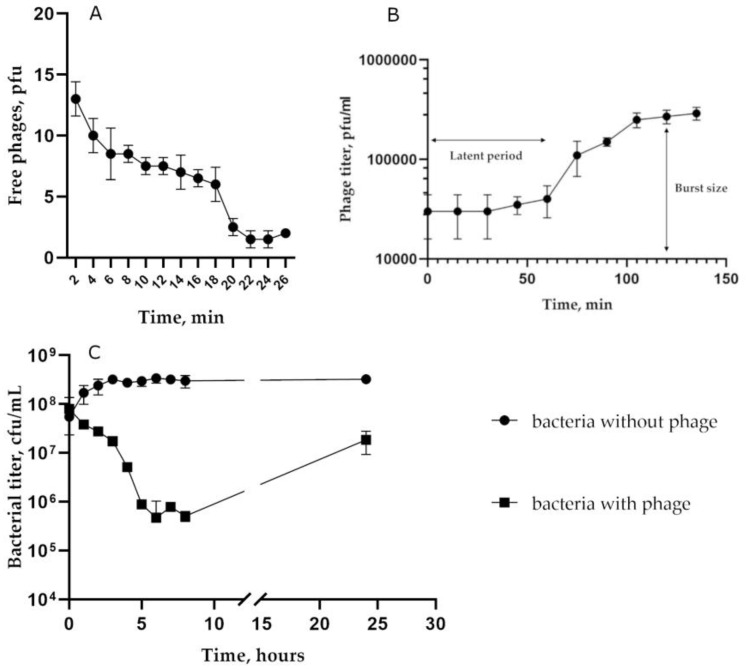
Biological properties of the phage St 134: (**A**) phage adsorption to host cells; (**B**) burst size experiments; (**C**) bacterial lytic curve.

To characterize biological properties of the phage St 134, the host strain *S. epidermidis* CEMTC 2058 was used (Figure 2). An adsorption experiment indicated that approximately 85% of St 134 phage particles attached to host cells within 22 min (Figure 2A). The one-step growth assay showed that the latent period for St 134 was ~60 min and that its burst size was only ~9 phage particles per infected cell (Figure 2B). The multistep bacterial killing experiments confirmed that St 134 has weak lytic activity (Figure 2C).

To determine the phage host range, 457 *Staphylococcus* strains of 24 various species were used (Table 2). Of these, 442 strains were obtained from clinical samples and 15 strains were from pets and animals living close to people. Of the tested strains, 204 were coagulase-positive *S. aureus* and *S. intermedius,* whereas 253 strains were coagulase-negative *Staphylococcus* spp. Of the coagulase-negative strains, 57 strains were St 134-sensitive, including 6 strains obtained from pets. The isolation year, geographic location, sample type, resistance to antibiotics, and GenBank ID of all St 134-sensitive staphylococcal strains are presented in Table S1. Most of the susceptible strains were *S. epidermidis* (Table 2), the other 24 strains that were sensitive to St 134 included three *S. haemolyticus* strains; all tested *S. capitis*, *S. caprae*, *S. casei*, *S. cohnii*, and *S. lugdunensis* strains; and single strains from other species (Table 2 and Table S1). As the number of tested *S. capitis*, *S. caprae*, *S. casei*, *S. cohnii*, and *S. lugdunensis* strains was small, it is not possible to conclude that these species are widely susceptible to St 134 infection (Table 2 and Table S1). So, St 134 infected various strains of 12 of the 21 tested coagulase-negative *Staphylococcus* species. However, no sensitive strains were found among *S. saprophyticus* and *S. warneri*, as well as coagulase-positive *S. aureus* and *S. intermedius*.

Unexpectedly, one coagulase-positive strain (CEMTC 3692) was shown to be susceptible to St 134 infection. The sequencing of its 16S rRNA gene fragment (1364 bp) indicated a high nucleotide identity (NI) with those of *Staphylococcus roterodami* (NI = 100%) and *Staphylococcus argenteus* (NI = 99.93%). These two species are part of the *S. aureus* complex and were isolated from a human foot infection and hip joint infection [47,48]. Despite the fact that St 134 could infect 13 *Staphylococcus* species of the 24 tested ones, this phage has a narrow host range; the highest proportion of sensitive strains was found among *S. epidermidis* and amounted to only 25% of them. 

### 3.3. Genome Characteristics and Comparative Analysis of St 134

**Figure 3:**
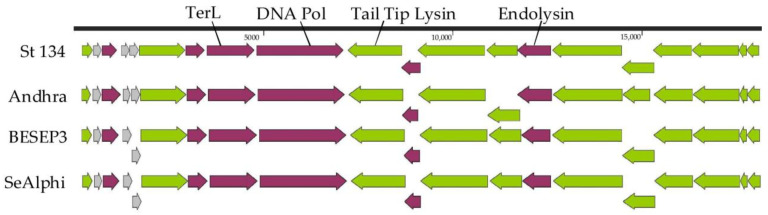
Genome maps of the St 134 phage and *Staphylococcus* phages from the *Andhravirus* genus. ORFs encoding structural and hypothetical proteins are marked with green and grey, respectively.

The St 134 genome is a double-stranded DNA of 18,275 bp (GenBank: KY471386). Twenty putative ORFs were found and nine of them are oriented in the forward direction, while eleven ORFs are in the opposite direction (Figure 3). Of these 20 ORFs, 17 ORFs encode proteins with predicted functions (Table S2) and only 3 ORFs encode hypothetical proteins. The first block of ORFs contains the genes encoding DNA polymerase; the terminase large subunit; several virion proteins, including virion-associated tail tip lysin; and three relatively small hypothetical proteins. The second block consists of genes encoding holin, endolysin, and structural proteins (Figure 3).

**Figure 4:**
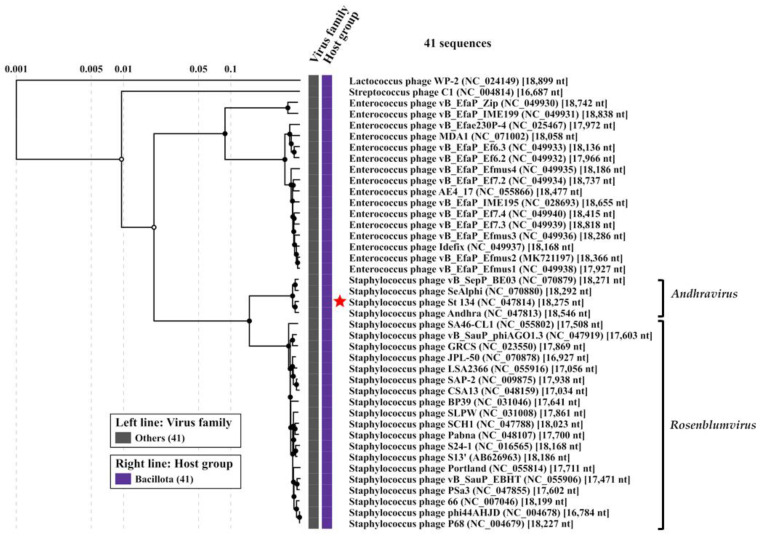
ViPTree analysis of the *Staphylococcus* phage St 134, which is marked with a red star. Black circles mark the nodes of the tree. Inner nodes, represented by black circles, are linked to an alignment of genomes that are included in its subtree, which is available on the ViPTree web server.

Comparative genome analysis indicated that the St 134 phage is a member of the *Andhravirus* genus (*Rountreeviridae* family). The highest similarity was found between St 134 and the *Staphylococcus* phage Andhra (NCBI: NC_047813, GenBank: KY442063) [49]; the nucleotide identity (NI) between these phages, which was calculated in the BioEdit 7.2.5 program [50], was 92.4%, meaning that these phages belong to different species within the same genus. The genomes of all known phages from the *Andhravirus* genus are similar in size (~18 kb), and their organization is similar too. All genomes contain 20 ORFs and are divided into the same two blocks according to the ORFs’ orientation (Figure 3). Most genes are orthologous and located in the same positions in the genomes of the *Andhravirus* species. The ViPTree analysis confirmed the taxonomy of St 134 (Figure 4). The similarity between St 134 and Andhra phages at the protein level was 97.5% (protein identity 95.9%). Notably, all members of the *Andhravirus* genus and its neighboring *Rosenblumvirus* genus infect *Staphylococcus* spp.

### 3.4. Phylogenetic Analysis of the St 134 Proteins

**Figure 5:**
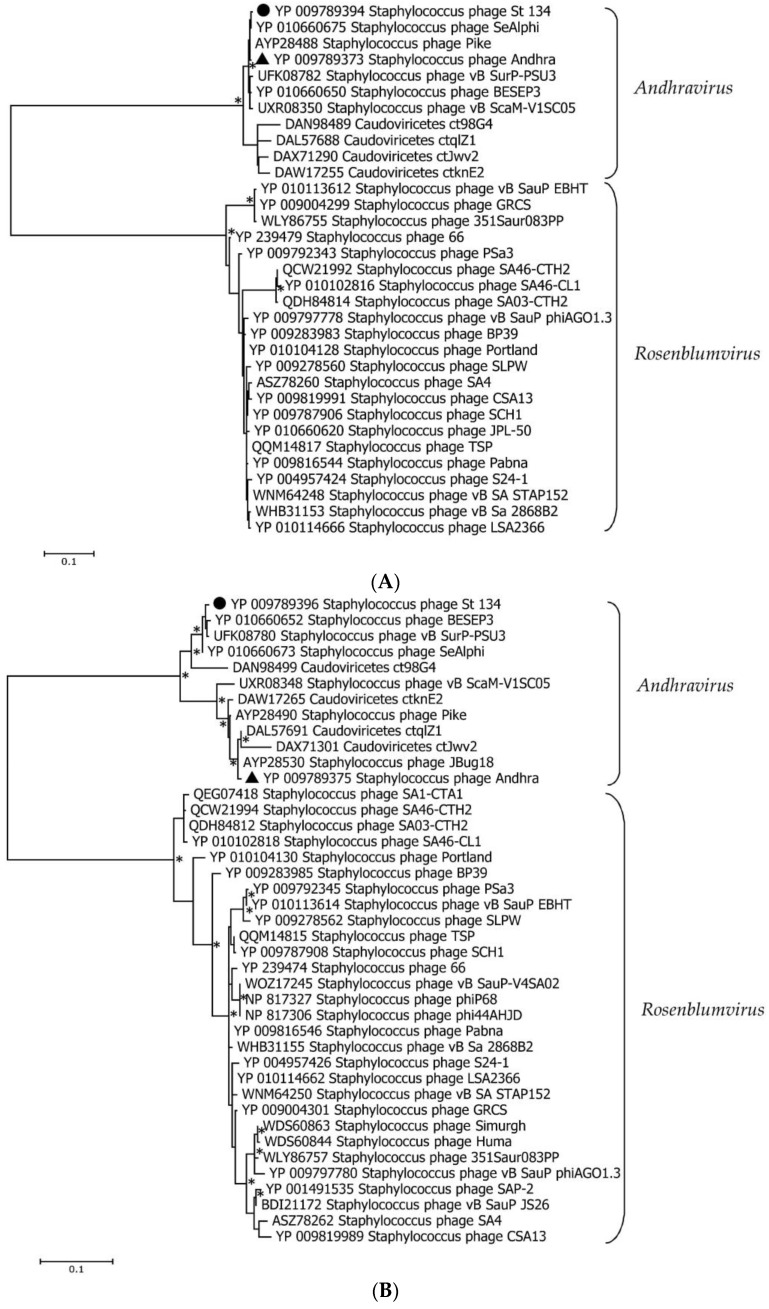

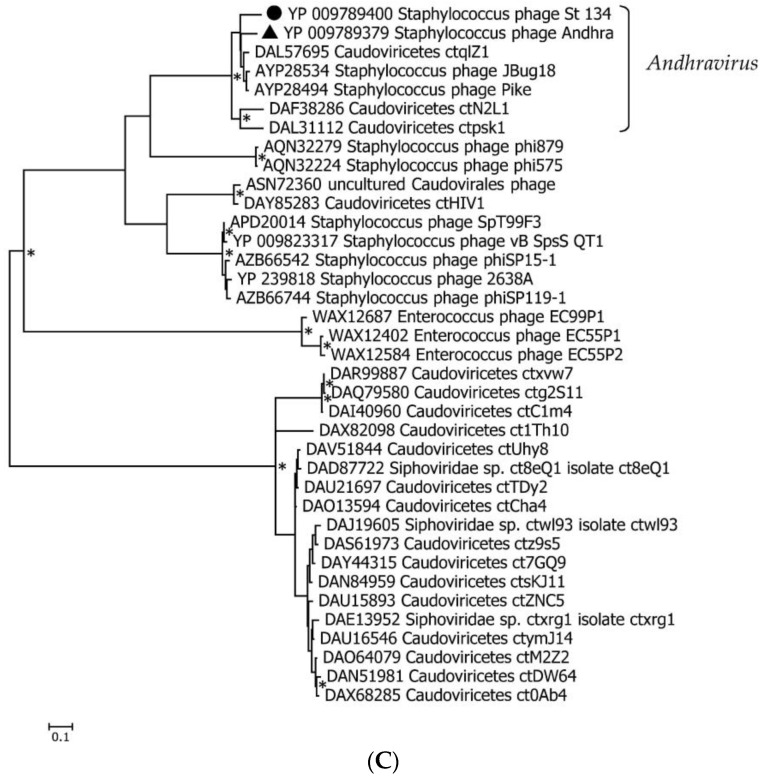
Maximum Likelihood phylogenetic trees of the St 134 terminase large subunit (**A**), tail tip lysin (**B**), and endolysin (**C**) generated using IQ-tree software. Corresponding sequences of the St 134 phage are marked with black circles; sequences of the *Staphylococcus* phage Andhra are marked with black triangles. Nodes with 95% statistical significance, calculated from 1000 ultrafast bootstrap replicates, are marked with asterisks. The scale bar represents the number of substitutions per site.

Phylogenetic analysis was performed for the St 134 terminase large subunit, tail tip lysin, and endolysin (Figure 5). In each case, the 40 most similar sequences were used for analysis in accordance with the E value of the BLASTp comparison. On the Maximum Likelihood (ML) phylogenetic tree of the St 134 terminate large subunit, phage sequences of the *Andhravirus* genus form a monophyletic, highly supported clade (Figure 5). Several unknown phage sequences from the metagenome-assembled genomes (MAGs) are grouped with this clade. The remaining sequences belong to the *Rosenblumvirus* genus and are clustered separately (Figure 5A).

The topology of the ML tree containing the St 134 tail tip lysin is similar to that of the terminase large subunit (Figure 5B). Analysis of the St 134 tail tip lysin using BLASTp demonstrated that it has 89% similarity (100% coverage) with the Andhra phage protein gp10 (tail tip lysin gp10, YP_009789375.1). This structural lysin contains an N-terminal glycosyl hydrolase (GyH) domain and a C-terminal cysteine, histidine-dependent amidohydrolases/peptidases (CHAP) domain. A clade of sequences of the genus *Andhravirus* is also formed with high reliability and divided into two subclades. The remaining sequences belong to the genus *Rosenblumvirus* and are grouped separately. As for the endolysin’s phylogeny, only four sequences from the known *Andhraviruses*, including the St 134 and Andhra phages, are found on the ML tree. St 134′s endolysin (LysSte134_1) sequence showed 81% similarity (100% coverage) with the Andhra phage protein gp14 (N-acetylmuramuramoyl-L-alanine amidase, YP_009789379). Three additional sequences from MAGs are grouped with them. The remaining sequences have a low level of similarity with the endolysin sequences from the *Andhravirus* genus (Figure 5C). 

### 3.5. In Silico Characterization of St 134 Lytic Enzymes

The putative endolysin LysSte134_1 consists of 295 amino acids (aa) with an isoelectric point (pI) of 9.21 and a predicted molecular weight of 35 kDa. The InterPro software package (https://www.ebi.ac.uk/interpro/search/sequence/, accessed on 21 August 2023) was used to determine LysSte134_1′s domains. It was shown that LysSte134_1 belongs to the superfamily of N-acetylmuramoyl-L-alanine amidase/peptidoglycan recognition proteins (Amidase/PGRP_sf) (IPR036505) and contains two domains. Its N-terminal domain is the N-acetylmuramoyl-L-alanine amidase-like type II (NALAA-2, EC 3.5.1.28, EC 3.5.1.28) domain (IPR002502). In the LysSte134_1 sequence, the NALAA-2-like catalytic domain (pfam01510) is located between 22 and 147 aa. The C-terminal region (190–295 aa) of LysSte134_1 has similarity with the SH3 domains (G3DSA:2.30.30.40).

**Figure 6:**
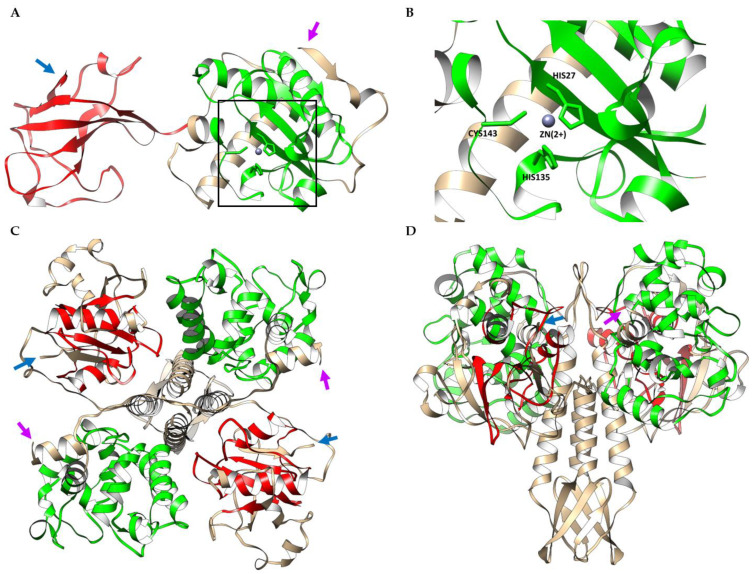
(**A**) Ribbon representation of the predicted 3D structure of the endolysin LysSte134_1 in complex with a Zn2+ ion; EAD and SH3 are marked in green and red, respectively. (**B**) Ribbon representation of the putative zinc binding site of LysSte134_1; His27, His135, and Cys143 are shown in stick form. (**C**) Ribbon representation (top view) of the predicted 3D dimer structure of the putative tail tip lysin LysSte134_2; GyH and CHAP are marked in green and red, respectively. (**D**) Ribbon representation (side view) of the predicted 3D dimer structure of LysSte134_2; GyH and CHAP are marked in green and red, respectively. The N- and C-terminus of the endolysin are marked with magenta and blue arrows, respectively. The molecular coordinates of the predicted 3D structures of St 134′s lytic enzymes were rendered using the UCSF Chimera molecular visualizer, version 1.15.

The putative structure of LysSte134_1 was predicted using AlphaFold2. It was confirmed that Lys Ste134_1 consists of two domains. The N-terminal globular core contains the predicted enzymatic NALAA-2-like domain that connects to the C-terminal globular SH3 domain via an unordered linker (Figure 6). The N-terminal NAALA-2-like domain contains four α-helices and one β-sheet; the C-terminal domain consists of two β-sheets interlinked by flexible linkers. The ligand-binding site of LysSte134_1 was predicted using a meta-server approach to protein-ligand binding site prediction, COFACTOR. This prediction indicated that the catalytic zinc ion (Zn^2+^) is coordinated with His27, His135, and Cys143 (Figure 6). 

The putative tail tip lysin LysSte134_2 contains 472 aa with a pI of 8.91 and predicted molecular weight of 51.4 kDa. HHpred, InterPro (https://www.ebi.ac.uk/interpro/search/sequence/, accessed on 21 August 2023), and the CDD database were used to identify its domain structure. It was indicated that LysSte134_2 belongs to the superfamily NLPC_P60 and contains two enzymatic domains: N-terminal GyH and C-terminal CHAP endopeptidase (IPR007921). The C-terminal domain is similar to CATH-Gene3D 3DSA:3.90.1720.10. The N-terminal GyH-like domain is located between 10 and 183 aa, whereas the CHAP domain (Pfam PF05257) is located between 351 and 439 aa. The cysteine, histidine-dependent amidohydrolases/peptidases (CHAP) hydrolyze peptidoglycans in the bacterial cell wall.

AlphaFold2 showed that the putative 3D structure of LysSte134_2 is similar to the Andhra gp10 protein [49,50,51]. In LysSte134_2, the N-terminal globular core contains the predicted enzymatic GyH-like domain and connects to the C-terminal globular CHAP domain via an ordered linker domain (middle linker). This linker domain consists of two α-helices and one β-sheet (Figure 6). Probably, LysSte134_2 forms a homodimer like gp10 of the Andhra phage (Figure 6). The N-terminal GyH-like domain contains eight α-helices; the C-terminal CHAP-like domain consists of two α-helices and one β-sheet. 

The solubility of putative recombinant LysSte134_1 and LysSte134_2 was assessed using the SoluProt and Protein-sol bioinformatics tools. SoluProt demonstrated that both putative enzymes have suitable solubility scores (>0.5) to be produced in *E. coli* cells. However, the analysis using Protein-sol indicated that while LysSte134_1 has adequate solubility scores (~0.45), LysSte134_2 has solubility scores that are insufficient for its production in *E. coli* cells (much less than 0.45).

### 3.6. Cloning, Production, and Purification of LysSte134_1 and LysSte134_2

**Figure 7:**
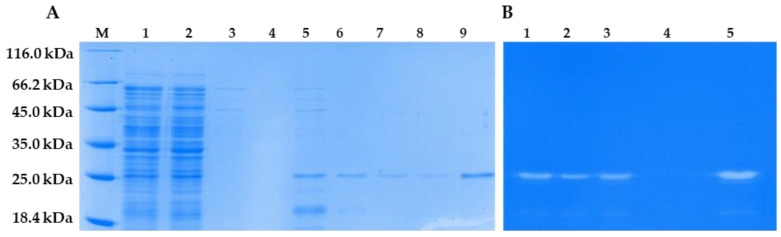
LysSte_1 purification and enzymatic activity. (**A**) 1—lysates of *E. col*i M15-pQE-60/LysSte134_1 cells producing recombinant LysSte134_1; 2—soluble cytoplasm of *E. coli* M15-pQE-60/LysSte134_1; 3—eluate after washing with buffer A; 4–9—eluate after elution with buffer A, which contains 50 mM, 100 mM, 150 mM, 200 mM, 250 mM and 350 mM imidazole, respectively. (**B**) Zymographic assay of LysSte_1 eluated with buffer A, which contained 100 mM, 150 mM, 200 mM, 250 mM, and 350 mM imidazole, respectively (Lines: 1–5). M—protein ladder Pierce™ Unstained Protein MW Marker #26610 (Thermofischer, Waltham, MA, USA).

The genes encoding LysSte134_1 and LysSte134_2 were inserted into the expression plasmid pQE-60. *E. coli* M15 cells were transformed with the constructed plasmids pQE-60-LysSte134_1 and pQE-60-LysSte134_2 to produce the recombinant proteins LysSte134_1 and LysSte134_2. Various growth conditions were tested to increase the solubility of the recombinant proteins. The localization and yield of LysSte134_1 and LysSte134_2 were evaluated using PAGE electrophoresis (Figure S2). LysSte134_1 was found in the soluble fraction of the cytoplasm only after the cultivation of *E. coli* M15/pQE-60-LysSte134_1 cells at 10 °C and its induction with 100 µM IPTG. LysSte134_1 was purified using Ni-NTA agarose from the soluble fraction of the cytoplasm, and its molecular weight was ~35 kDa, which corresponded to the predicted weight (Figure 7). The purity of LysSte134_1 after chromatography was evaluated using PAGE, and it was ~95% (Figure 7). The yield of LysSte134_1 production was 2 mg from 1 L of cell culture after all stages of purification and dialysis. The purified LysSte134_1 was stored at a concentration of more than 1 mg/mL in S-buffer (50 mM Tris-HCl pH 7.5, 300 mM NaCl) at 4 °C.

Unlike LysSte134_1, the recombinant LysSte134_2 was insoluble under all cultivation conditions. Several attempts were made to refold LysSte134_2 from the insoluble cytoplasm fraction as described previously [52]. During dialysis, LysSte134_2 precipitated when the concentration of urea in the re-coagulation buffer was below 1 M. Thus, a purified soluble preparation of this protein was not obtained, and the insoluble fraction of the cytoplasm was used to assess the enzymatic activity of this protein.

### 3.7. Enzymatic Activity of LysSte134_1 and LysSte134_2

**Figure 8:**
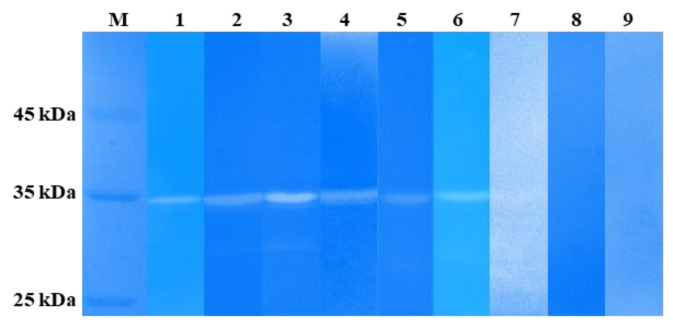
Zymographic analysis of recombinant LysSte134_1. PAGE (12,5%) containing 0.5 mg of peptidoglycans from *S. aureus* CEMTC 675 (1), *S. aureus* CEMTC 1685 (2), *S. epidermidis* CEMTC 2058 (3), *S. epidermidis* CEMTC 2043 (4), *S. haemolyticus* CEMTC 3753 (5), *S. warneri* CEMTC 2062 (6), *S. warneri* CEMTC 4154 (7), *S. saprophyticus* CEMTC 3872 (8), and *S. saprophyticus* CEMTC 6829 (9), stained with methylene blue. M—protein ladder Pierce™ Unstained Protein MW Marker #26610 (Thermofischer, Waltham, MA, USA).

The lytic activity of LysSte134_1 and LysSte134_2 was assessed by zymographic assay using the cell wall peptidoglycans of various *Staphylococcus* strains, both sensitive and insensitive to St 134. In addition, all used strains were isolated from clinical samples. It was shown that LysSte134_1 hydrolyzed peptidoglycan from the cell wall of *S. epidermidis* CEMTC 2058, which is a host strain of St 134 phage (Figure 7). In addition, LysSte134_1 was able to digest peptidoglycans from other coagulase-negative strains, namely *S. epidermidis* CEMTC 2043, *S. haemolyticus* CEMTC 3413 (both are MDR strains sensitive to St 134), and *S. warneri* CEMTC 2062 (weak activity) (Figure 8). However, it could not cleave the peptidoglycans isolated from the cell wall of *S. warneri* CEMTC 4154 and both tested *S. saprophyticus* strains CEMTC 3872 and CEMTC 6829. The parental St 134 phage cannot infect all tested *S. warneri* and *S. saprophyticus* strains (Table 2 and Table S1). 

In addition, LysSte134_1 hydrolyzed peptidoglycans from the cell wall of the coagulase-positive strains *S. aureus* CEMTC 675 (strain with MDR) and *S. aureus* CEMTC 1685 (VRSA) (Figure 8). Both *S. aureus* strains are not susceptible to St 134 infection. The obtained results demonstrated that LysSte134_1 was able to hydrolyze peptidoglycans from various pathogenic *Staphylococcus* strains despite their sensitivity to its parental phage St 134 (Figure 8). 

### 3.8. Anti-Staphylococcal Activity of LysSte134_1

**Figure 9:**
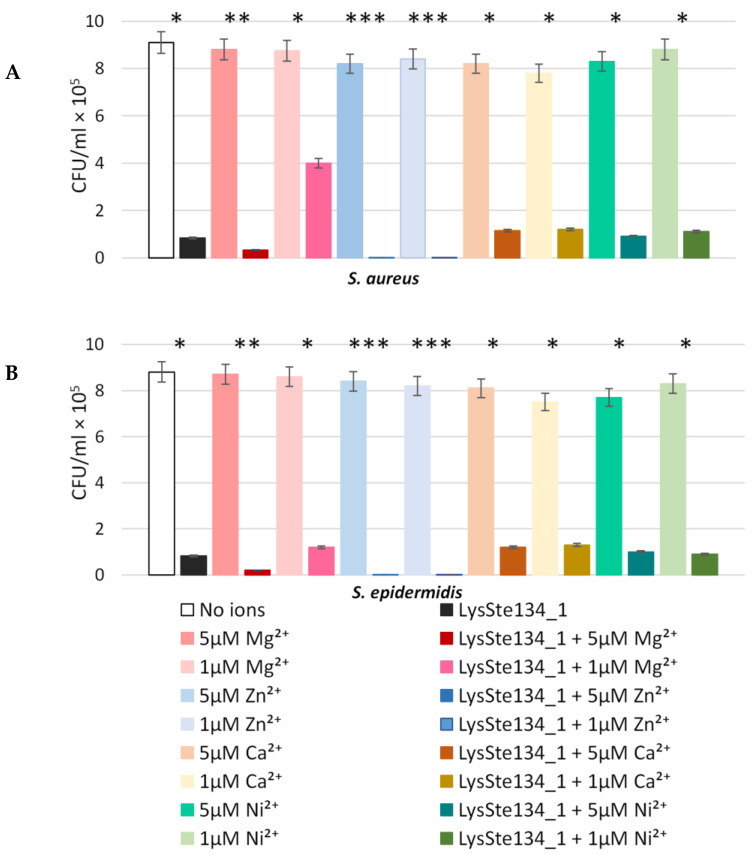
Antibacterial activity of LysSte134_1 against S. aureus CEMTC 1685 (**A**) and *S. epidermidis* CEMTC 2043 (**B**). LysSte134_1 (100 µg/mL) in R buffer (50 mM Tris-HCl; pH 8.0) was added to staphylococcal cells with or without CaCl_2_, MgSO_4_, ZnCl_2_, or NiSO_4_ at different concentrations, and the obtained suspensions were incubated for two hours at 37 °C before aliquots of the suspensions were plated on LB agar plates. The next day, colonies were counted. Cell cultures with only CaCl_2_, MgSO_4_, ZnCl_2_, or NiSO_4_ were used as controls. Experiments were performed in triplicate. *** *p* < 0.001, ** *p* < 0.01, * *p* < 0.05.

The antibacterial activity of LysSte134_1 was assessed against planktonic cultures of *S. aureus* CEMTC 1685 and *S. epidermidis* CEMTC 2043. It was demonstrated that the treatment of *S. aureus* CEMTC 1685 or *S. epidermidis* CEMTC 2043 cells with LysSte134_1 (25 µg/mL) resulted in a 50-fold decrease in their CFUs (Figure 9). Then, the effect of different concentrations of bivalent ions (Ca^2+^, Mg^2+^, Zn^2+^, and Ni^2+^) on the effectivity of LysSte134_1 was studied. When *S. aureus* CEMTC 1685 and *S. epidermidis* CEMTC 2043 were treated with LysSte134_1 that was supplemented with ZnCl_2_ at concentrations of 1 µM and 5 µM, the lytic activity of LysSte134_1 against these strains increased up to 100-fold and 1000-fold, respectively (Figure 9). Notably, Ca^2+^, Mg^2+^, and Ni^2+^ did not significantly affect the hydrolytic activity of LysSte134_1 against both tested *Staphylococcus* strains. The results indicated that LysSte134_1 is a Zn2^+^-dependent enzyme, similar to other amidases from the NALAA-2 group that cleave the amide linkage between N-acetylmuramoyl and L-amino acids in the bacterial cell wall [53].

### 3.9. Biofilm Disruption Activity of LysSte134_1

**Figure 10:**
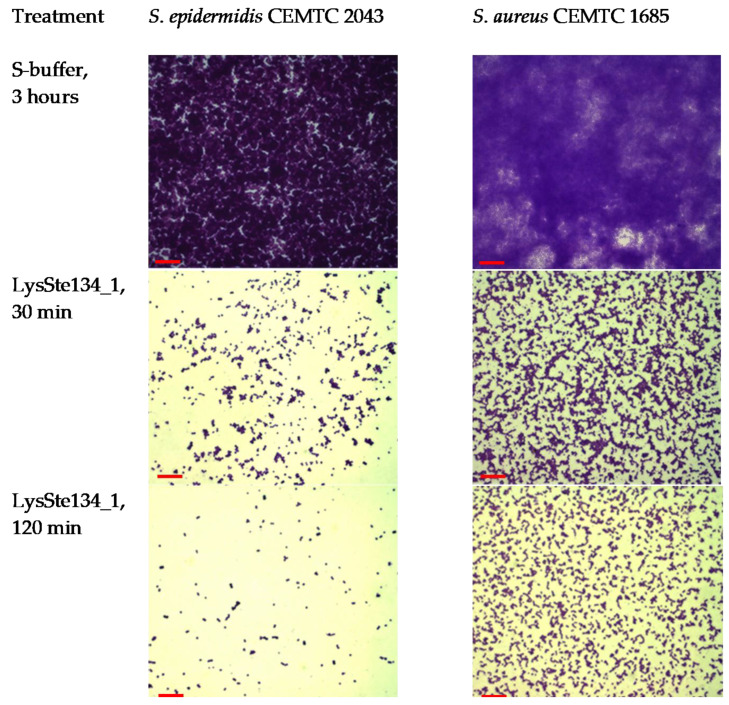
LysSte134_1 activity against biofilms that were formed by *S. epidermidis* CEMTC 2043 and *S. aureus* CEMTC 1685. Bacterial cells and matrix were stained with methyl violet. After the treatment of biofilms with LysSte134_1, remnants of a biofilm matrix are observed, whereas, in biofilm samples treated with S-buffer, an extensive biofilm matrix is visible, densely filled with staphylococci. Red scale bar—10 µm.

LysSte134_1 activity against staphylococcal biofilms was evaluated using biofilms formed by *S. epidermidis* CEMTC 2043 and *S. aureus* CEMTC 1685. Biofilms were grown on the surface of coverslips in LB medium. When the biofilms had formed, they were treated with purified LysSte134_1 at a concentration of 25 μg/mL in PBS. Sterile S-buffer was added as a control. The biofilms with LysSte134_1 were incubated at 37 °C for 30 min and 120 min, while the biofilms with PBS were incubated for ~180 min. In the *S. epidermidis* biofilm treated with LysSte134_1, only small remnants of the biofilm’s matrix and cells were observed after incubation for 30 min (Figure 10). LysSte134_1 was active against the biofilm formed by *S. aureus* cells to a lesser extent than in case of *S. epidermidis*; however, even this biofilm was substantially disrupted within 30 min. In control biofilms, a tight matrix of stained bacteria was observed (Figure 10).

## 4. Discussion

Phage-encoded lytic enzymes are promising agents against drug-resistant bacterial pathogens. In this study, a new podophage St 134 was described and functional and structural analyses, in silico, of its recombinant hydrolases LysSte134_1 and LysSte134_2 were performed. Considering the size and organization of the St 134 genome, as well as its nucleotide identity to the nearest genomes, it was concluded that St 134 is a typical member of the *Andhravirus* genus (*Rountreeviridae* family). Besides the *Staphylococcus* phages Andhra [49] and St 134, this genus currently contains several other described phages, namely JBug18, Pike, Pontiff vBSepPBE03, SeAlphi, and vB_SurP-PSU3 [54,55,56,57]. The bacterial hosts of all known members of the *Andhravirus* genus are coagulase-negative *Staphylococcus* spp.; most of these phages infect *S. epidermidis*, whereas vB_SurP-PSU3 infects *Staphylococcus ureilyticus.* Notably, phages from the neighbour genus *Rosenblumvirus* (*Rountreeviridae* family) can infect coagulase-positive *S. aureus* [54,58,59,60,61,62,63], with the exception of the *S. aureus* phage CSA13, which can additionally infect some coagulase-negative *Staphylococcus* spp. [64]. The studied phage St 134, being a *S. epidermidis* phage with a narrow host range, demonstrated the ability to infect 11 other coagulase-negative species, including *S. ureilyticus*. In addition, one coagulase-positive strain of the *S. aureus* complex was sensitive to St 134. As far as we know, St 134 is the only phage of the *Andhravirus* genus that can infect a coagulase-positive *Staphylococcus* strain.

In the St 134 genome, the genes encoding its structural lysin and endolysin were identified. The first gene encodes the tail tip lysin (Genkbank: AQT25384), which cleaves cell walls’ peptidoglycans after phage adsorption, allowing the introduction of phage DNA into the cell. The second gene encodes endolysin (GenBank: AQT25388), which is produced in the host cell’s cytoplasm and ensures the release of phage progeny from cells. The endolysin LysSte134_1 contains an N-terminal NALAA-2-like catalytic domain and C-terminal SH3 domain. The tail tip lysin LysSte134_2 consists of an N-terminal GyH-like domain connected to a C-terminal CHAP domain via a linker domain (structured linker), which forms the dimer structure of LysSte134_2.

Phylogenetic analysis indicated that the well-characterized hydrolases of the Andhra phage are most similar to the studied LysSte134_1 and LysSte134_2: Andhra_gp10, which is a homologue of LysSte134_2, and Andhra_gp14, which is a homologue of LysSte134_1 [49,51]. Notably, LysSte134_1 showed antibacterial activity against both *S. aureus* and *S. epidermidis* planktonic cultures, whereas the Andhra_gp14 endolysin demonstrated little or even no activity against *S. aureus* [49]. Moreover, the ability of LysSte134_1 to disrupt biofilms formed by *S. aureus* and *S. epidermidis* was confirmed. Such differences are probably due to the fact that although Andhra_gp14 and LysSte134_1 are similar proteins, their similarity rate is only 80%, while the total protien similarity between these phages is 97.5%. Notably, the largest number of substitutions in both endolysins is located in the NALAA-2 and SH3 domains, which probably leads to an increase in the spectrum of the lytic activity of LysSte134_1. Unfortunately, the hydrolytic activity of LysSte134_1 against peptidoglycans from *S. intermedius* and its planktonic culture was not tested, which is a limitation of this study. This should be carried out in the future.

Both *S. aureus* and *S. intermedius* are coagulase-positive staphylococci; they belong to different species complexes. Usualy, strains from the *S. aureus* complex cause various severe infections in humans, including meningitis and sepsis, whereas strains from the *S. intermedius* complex are mainly the causative agents of veterinary infections and can be transmitted to humans through contact with animals [53,65,66,67]. The high lytic activity of LysSte134_1 against planktonic cultures of *S. aureus* and the biofilms that were formed by *S. aureus* is an advantage of the LysSte134_1 endolysin over the Andhra_gp14 and Andhra_gp10 enzymes. Expectedly, LysSte134_1 showed higher efficacy against *S. epidermidis* cells and their biofilms than against *S. aureus* ones. Although it is believed that *S. epidermidis* is a less virulent pathogen than *S. aureus*, in recent years the proportion of *S. epidermidis* strains with MDR is significantly higher than that for *S. aureus,* at least in some regions [3]. In addition, *S. epidermidis*, as a normal skin commensal, is capable of forming biofilms, which is a serious problem in intensive care units and especially in surgery.

In conclusion, taking into consideration the hydrolytic activity of LysSte134_1 against both coagulase-positive *S. aureus* and coagulase-negative *Staphylococcus* spp., we can consider this endolysin an antistaphylococcal agent that is able to remove staphylococcal biofilms. However, further investigations are required to determine the range of *Staphylococcus* species that can be lysed using LysSte134_1. In addition, these phage lytic enzymes can be used in combination with antibiotics, as they can digest and destroy the biofilm matrix, which enables antibiotics to reach their target cells and, in turn, can lead to a decrease in the dosage of antibiotics used. With a low risk of resistance development [68,69,70], endolysins can be administrated both as an independent antimicrobial agent and in combination with phages to treat MDR and, especially, pandrug-resistant bacteria.

## Figures and Tables

**Table 1:** Primers used for cloning and sequencing.

Primer Title	Primer Sequence
endolysin_StE134_1U	5′-GGGAACCATGGGAATGAAAAATATTTATTCAAACCACATTAAAGG-3′
endolysin_StE134_1L	5′-GGGATGGATCCGTCAACCTCCAATTTTCCCCAAAGA-3′
endolysin_StE134_2U	5′-CCGAATCATGAATGATAAAGAAAAAATTGACAAGTTTATACAT-3′
endolysin_StE134_2L	5′-CCCAAGGATCCCTTAATACCACTAAAAAACATAATATTATCCCCTG-3′
pQE60-SeqU	5′-GATTCAATTGTGAGCGGATAAC-3′
pQE60_SeqL	5′-ATCCAGATGGAGTTCTGAGGTC-3′

**Table 2:** *Staphylococcus* strains tested for their sensitivity to the St 134 phage.

№	Species	Number of Tested Strains	Number of Strains Sensitive to St 134
**Coagulase-positive *Staphylococcus* spp.**			
1	*S. aureus*	178	0
2	*S. aureus* complex (*S. roterodami*/*S. argenteus*)	1	1
3	*S. intermedius*	25	0
**Coagulase-negative *Staphylococcus* spp.**			
4	*S. epidermidis*	132	33 (25%)
5	*S. auricularis*	5	1
6	*S. borealis*	2	0
7	*S. capitis*	4	4
8	*S. caprae*	5	5
9	*S. carnosus*	2	0
10	*S. casei*	2	2
11	*S. coagulans*	8	1
12	*S. cohnii*	5	2
13	*S. devriesei*	4	1
14	*S. equorum*	4	2
15	*S. felis*	6	0
16	*S. haemolyticus*	28	3 (10.7%)
17	*S. hominis*	22	0
18	*S. lugdunensis*	2	2
19	*S. pasteuri*	1	0
20	*S. saprophyticus*	2	0
21	*S. simulans*	8	0
22	*S. succinus*	1	0
23	*S. ureilyticus*	1	1
24	*S. warneri*	9	0
	**Total**	**457**	**68**

## Data Availability

The St 134 phage genome was submitted to GenBank (http://www.ncbi.nlm.nih.gov, accessed on 15 May 2020) under the accession number KY471386.

## Supplementary Materials

The following supporting information can be downloaded at https://www.mdpi.com/article/10.3390/v16030385/s1, Table S1: *Staphylococcus* spp. strains sensitive to the St 134 phage. Table S2: open reading frames (ORFs) found in the genome of the St 134 phage. Figure S1: electron micrographs of St 134 phage particles negatively stained with 1% uranyl acetate. Scale bar was 50 nm. Figure S2: SDS PAGE (A) 1—lysates of *E. coli* M15-pQE-60/LysSte134_1 cells, producing recombinant LysSte134_1; 2—insoluble cytoplasm of *E. coli* M15-pQE-60/LysSte134_1; 3—soluble cytoplasm of *E. coli* M15-pQE-60/LysSte134_1. (B) 1—lysates of *E. coli* M15-pQE-60/LysSte134_2 cells, producing recombinant LysSte134_2; 2—insoluble cytoplasm of *E. coli* M15-pQE-60/LysSte134_1; 3—soluble cytoplasm of *E. coli* M15-pQE-60/LysSte134_2.
