# Supplementary material for: Bacteriophage vB_SepP_134 and Endolysin LysSte_134_1 as Potential Staphylococcus-Biofilm-Removing Biological Agents
Source: Viruses. 2024 Feb 29;16(3):385. doi: 10.3390/v16030385 (PMC10975630; doi:10.3390/v16030385)
Supplement: Supplementary file 1 [file viruses-16-00385-s001.zip › Figure S1 S2 revised.pdf]

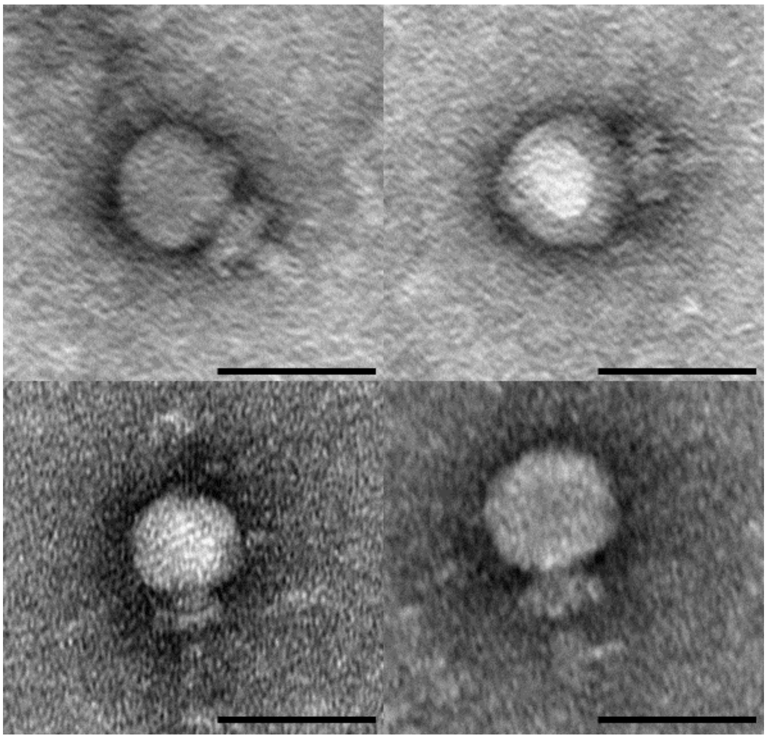

Figure S1: Electron micrographs of St_134 phage particles negatively stained with 1% uranyl acetate. Scale bar was 50 nm.

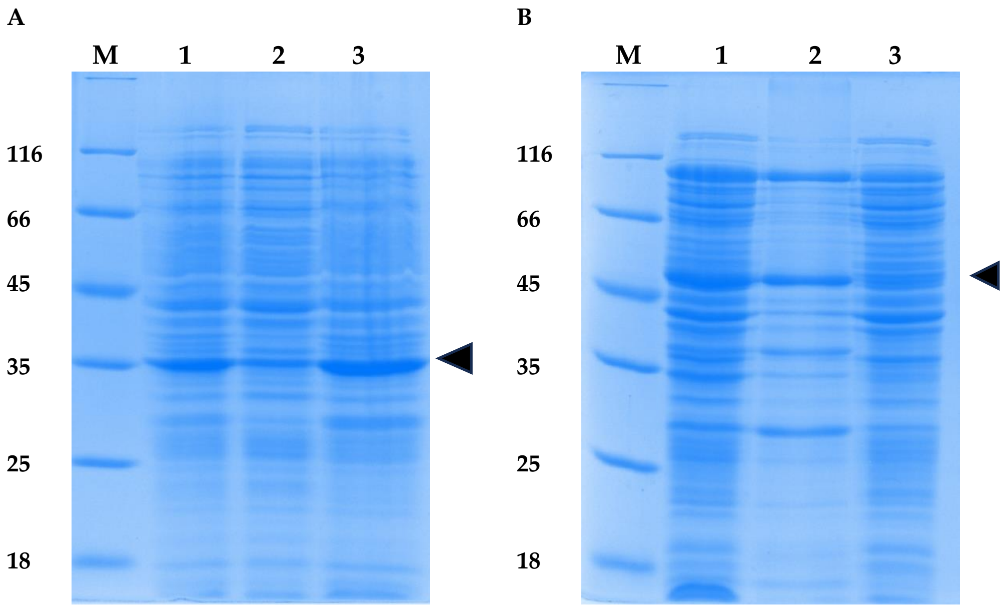

Figure S2: SDS PAGE (A): 1 - lysates of *E. coli* M15-pQE-60/LysSte134_1 cells, producing recombinant LysSte134_1; 2 – insoluble cytoplasm of *E. coli* M15- pQE-60/LysSte134_1; 3 –soluble cytoplasm of *E. coli* M15- pQE-60/LysSte134_1. (B): 1 - lysates of *E. coli* M15-pQE-60/LysSte134_2 cells, producing recombinant LysSte134_2; 2 – insoluble cytoplasm of *E. coli* M15- pQE-60/LysSte134_2; 3 – soluble cytoplasm of *E. coli* M15- pQE-60/LysSte134_2. M- protein ladder 26610 Pierce ™ Unstained protein (Thermo Fisher Scientific, Waltham, MA, USA).
