# Supplementary material for: Bacteriophage vB_SepP_134 and Endolysin LysSte_134_1 as Potential Staphylococcus-Biofilm-Removing Biological Agents
Source: Viruses. 2024 Feb 29;16(3):385. doi: 10.3390/v16030385 (PMC10975630; doi:10.3390/v16030385)
Supplement: Supplementary file 1 [file viruses-16-00385-s001.zip › Table S1 Table S2 revised.pdf]

Table S1. *Staphylococcus spp.* strains sensitive to the St_134 phage

№	CEMTC* number	Collection date	Sample source	Coordinates	Antibiotic resistance	Aminoglycosides	β-lactams	Fluoroquinolones	Glycopeptides	Lincosamides	Macrolides	Oxazolidinones	Phenicol	Sulfonamides	Tetracyclines	GenBank Accession number
***S. aureus* complex (*S. roterodami*/*S. argenteus*) (1/1)**																
1	3692	May 2019	periprosthetic joint infection	55°02′15″N 82°55′39″E	S											OR856624
***S. auricularis* (1/5)**																
2	2738	Sep 2016	ear swab	54°51′02″N 83°06′16″E	R					DA						MZ014399
***S. capitis* (4/4)**																
3	1590	Apr-2013	urine	54°51′02″N 83°06′16″E	S											MZ014405
4	1680	Nov-2011	nasal swab	54°51′02″N 83°06′16″E	S											MZ014406
5	2904	Feb-2017	ear swab	54°51′02″N 83°06′16″E	S											MZ014411
6	3590	Jan-2019	skin	54°51′02″N 83°06′16″E	S											MZ014407
***S. caprae* (5/5)**																
7	1849	Apr-2014	skin, auricle	54°51′02″N 83°06′16″E	S											MZ014408
8	2305	Dec-2015	periprosthetic joint infection	55°02′15″N 82°55′39″E	S											MZ014409
9	2739	Sep-2016	skin, auricle	54°51′02″N 83°06′16″E	S											MZ014410
10	3411	Jun-2018	skin	54°51′02″N 83°06′16″E	S											MZ014412
11	3440	Aug-2018	skin, psoriasis	54°51′02″N 83°06′16″E	S											MZ014413
***S. casei* (2/2)**																
12	1626	Apr-2013	feces	54°51′02″N 83°06′16″E	S											MZ014416
13	2931**	Mar-2017	guinea pig, oral cavity	54°56′20″N 83°11′00″E	S											MW979954
***S. coagulans* (1/8)**																
14	3359**	Mar-2018	skin, dog	54°58′38″N 83°02′42″E	S											MW979970
***S. cohnii* (2/5)**																
15	2687	Jul-2016	feces	55°02′06″N 82°55′56″E	S											MZ014418
16	3694**	May-2019	chicken poop	52°46′N 82°37′E	S											MW969510
***S. devriesei* (1/4)**																
17	700	Apr-2010	urine	54°51′02″N 83°06′16″E	R						E					MZ014419
***S. epidermidis* (33/132) 25%**																
18	301	Jul-2009	skin, outpatient	55°03′N 82°57′E	S											OR856620
19	1380	Sep-2011	dental plaque	54°51′02″N 83°06′16″E	S											MZ049534
20	1548	Feb-2013	diabetic	55°02′13″N	R		FOX				E					MZ027385

			foot ulcer	82°53′00″E												
21	1683	Nov-2013	throat swab	54°51′02″N 83°06′16″E	R						E					MZ040900
22	1795	Mar-2014	bronchial mucus	54°51′02″N 83°06′16″E	S											OR856622
23	1821	Apr-2014	diabetic foot ulcer	55°02′13″N 82°53′00″E	R					DA						MZ040881
24	2043	Jan-2015	nasal swab, outpatient SR1	54°51′02″N 83°06′16″E	MDR		CFM	CIP, LEV	VA		E		C			MZ040893
25	2051	Jan-2015	throat swab, outpatient ZSG	54°51′02″N 83°06′16″E	R						E					MZ040901
26	2058***	Jan-2015	joint fluid	55°02′15″N 82°55′39″E	R		P									OR856623
27	2079	Feb-2015	surgical wound	54°58′60″N 82°54′52″E	R		FOX				E					MZ027387
28	2257	Oct-2015	dental plaque	55°02′32″N 82°57′06″E	S											PP264245
29	2283	Nov-2015	vaginal smear	54°51′02″N 83°06′16″E	S											MZ047210
30	2286	Nov-2015	vaginal smear	54°51′02″N 83°06′16″E	R	CN					E					MZ040911
31	2831	Jan-2017	vaginal smear	54°58′58″N 83°54′49″E	R						E					MZ040914
32	2889	Jan-2017	skin, auricle	54°51′02″N 83°06′16″E	MDR	AK	FOX				E					MZ027358
33	2908	Feb-2017	skin, auricle	54°51′02″N 83°06′16″E	S											MZ723060
34	2914	Feb-2017	nasal boil	54°51′02″N 83°06′16″E	S											MZ047208
35	2922	Feb-2017	skin, auricle	54°51′02″N 83°06′16″E	R	CN					E					MZ040905
36	3054	Oct-2017	surgical wound	54°59′10″N 82°54′55″E	MDR	CN	FOX			DA	E					MZ027389
37	3441	Aug-2018	skin, psoriasis	54°51′02″N 83°06′16″E	S											MZ049532
38	3463	Oct-2018	throat swab	54°51′02″N 83°06′16″E	S											MZ047207
39	3491	Oct-2018	nasal swab	54°51′02″N 83°06′16″E	R						E					MZ040898
40	3719	Jul-2019	feces	54°51′02″N 83°06′16″E	S											PP264246
41	3912	Jan-2020	bronchial mucus	54°51′02″N 83°06′16″E	R						E					MZ040903
42	4380	May-2021	throat swab	54°51′02″N 83°06′16″E	R	AK, CN	P, FOX									OR856625
43	4411	Jun-2021	throat swab	54°51′02″N 83°06′16″E	R	CN										OR856626
44	4422	Jun-2021	throat swab	54°51′02″N 83°06′16″E	R	AK					E					OR856627
45	4429	Jun-2021	throat swab	54°51′02″N 83°06′16″E	R			LEV								PP264247
46	4430	Jun-2021	throat swab	54°51′02″N 83°06′16″E	MDR		P					LNZ		SXT	TE	OR640305
47	4436	Jul-2021	throat swab	54°51′02″N 83°06′16″E	R	AK, CN										OR856629
48	4444	Jul-2021	throat swab	54°51′02″N 83°06′16″E	R	AK, CN										OR856630

49	4465	Nov-2021	throat swab	54°51′02″N 83°06′16″E	MDR	AK	FOX			DA	E					
50	6765	Jun-2022	urine	55°03′N 82°57′E	R		P, FOX				E					OR856632
S. equorum **(2/4)**																
51	3183**	Sep-2017	cloaca flush, red-headed duck	53°44′43″N 77°52′54″E	S											MW969511
52	3636**	Feb-2019	vaginal smear, dog	54°58′38″N 83°02′42″E	S											MW979971
S. haemolyticus **(3/28) 10.7%**																
53	1437**	Nov-2012	excrement, chinchilla	54°50′57″N 83°06′13″E	MDR	CN	FOX				E					MW979956
54	1700	Dec-2013	diabetic foot ulcer	55°02′13″N 82°53′00″E	MDR	CN	FOX				E					MZ027398
55	3413	Jul-2018	oral mucosa, ventilation	54°51′07″N 83°05′29″E	MDR	AK, CN	FOX				E					MZ027399
S. lugdunensis **(2/3)**																
56	4459	Jul-2021	nasal swab	54°51′02″N 83°06′16″E	R		FOX									OQ346157
57	4461	Jul-2021	nasal swab	54°51′02″N 83°06′16″E	S											OQ346157
S. ureilyticus **(1/1)**																
58	1966	Oct-2014	skin, crack between fingers	54°51′02″N 83°06′16″E	MDR		P	CIP, LEV		DA						OQ346151

The number of St_134-sensitive strains among tested strains is indicated in parentheses.

*– Collection of Extremophile Microorganisms and Type Cultures (CEMTC) of ICBFM SB RAS; **– strains isolated from pets; ***– host strain for the St_134 phage.

Abbreviations: MDR – Multi Drug Resistance; R – Resistance; S – Sensitive; AK – Amikacin; C - Chloramphenicol; CIP - Ciprofloxacin, CN – Gentamicin; DA – Clindamycin; FOX – Cefoxitin; E – Erytromycin; LEV – Levofloxacin; LNM – Lincomycin; P – Penicillin; SXT - Trimethoprim/sulfamethoxazole; TE – Tetracycline; VA – Vancomycin.

Table S2. Open reading frames (ORFs) found in the genome of the St_134 phage

ORF	Location	Strand	Function
1	St_134_131_499	+	tail tip protein
2	St_134_516_731	+	hypothetical protein
3	St_134_746_1138	+	single stranded DNA-binding protein
4	St_134_1263_1472	+	hypothetical protein
5	St_134_1476_1721	+	hypothetical protein
6	St_134_1711_2940	+	receptor binding anchor protein; hydrolase
7	St_134_2951_3454	+	5′ terminal protein
8	St_134_3513_4772	+	terminase
9	St_134_4830_7121	+	DNA polymerase
10	St_134_8655_7234	-	tail tip lysin

11	St_134_9070_8648	-	holin
12	St_134_10844_9078	-	tail knob protein
13	St_134_11707_10904	-	head fiber (Fib)protein
14	St_134_12595_11708	-	endolysin
15	St_134_14461_12632	-	receptor binding protein
16	St_134_15310_14474	-	tail stem/collar protein
17	St_134_16310_15303	-	portal protein
18	St_134_17548_16331	-	major capsid protein
19	St_134_17751_17560	-	capsid lining protein
20	St_134_18084_17764	-	portal-proximal core protein
